# Bayesian Network Expansion Identifies New ROS and Biofilm Regulators

**DOI:** 10.1371/journal.pone.0009513

**Published:** 2010-03-03

**Authors:** Andrew P. Hodges, Dongjuan Dai, Zuoshuang Xiang, Peter Woolf, Chuanwu Xi, Yongqun He

**Affiliations:** 1 Center for Computational Medicine and Bioinformatics, University of Michigan, Ann Arbor, Michigan, United States of America; 2 Department of Environmental Health Sciences, University of Michigan, Ann Arbor, Michigan, United States of America; 3 Unit for Laboratory Animal Medicine, University of Michigan, Ann Arbor, Michigan, United States of America; 4 Department of Chemical Engineering, University of Michigan, Ann Arbor, Michigan, United States of America; 5 Department of Biomedical Engineering, University of Michigan, Ann Arbor, Michigan, United States of America; 6 Department of Microbiology and Immunology, University of Michigan, Ann Arbor, Michigan, United States of America; East Carolina University, United States of America

## Abstract

Signaling and regulatory pathways that guide gene expression have only been partially defined for most organisms. However, given the increasing number of microarray measurements, it may be possible to reconstruct such pathways and uncover missing connections directly from experimental data. Using a compendium of microarray gene expression data obtained from *Escherichia coli*, we constructed a series of Bayesian network models for the reactive oxygen species (ROS) pathway as defined by EcoCyc. A consensus Bayesian network model was generated using those networks sharing the top recovered score. This microarray-based network only partially agreed with the known ROS pathway curated from the literature and databases. A top network was then expanded to predict genes that could enhance the Bayesian network model using an algorithm we termed ‘BN+1’. This expansion procedure predicted many stress-related genes (e.g., *dusB* and *uspE*), and their possible interactions with other ROS pathway genes. A term enrichment method discovered that biofilm-associated microarray data usually contained high expression levels of both *uspE* and *gadX*. The predicted involvement of gene *uspE* in the ROS pathway and interactions between *uspE* and *gadX* were confirmed experimentally using *E. coli* reporter strains. Genes *gadX* and *uspE* showed a feedback relationship in regulating each other's expression. Both genes were verified to regulate biofilm formation through gene knockout experiments. These data suggest that the BN+1 expansion method can faithfully uncover hidden or unknown genes for a selected pathway with significant biological roles. The presently reported BN+1 expansion method is a generalized approach applicable to the characterization and expansion of other biological pathways and living systems.

## Introduction

In this study, we explore how a biological pathway can be defined, and identify a set of methods to automatically learn a pathway from experimental data. Although many biological pathways have been described in the literature, these pathways likely represent only a small portion of the known underlying network of interactions. Recently, such pathway representations have been systematized in databases such as EcoCyc [1], RegulonDB [2], and KEGG [3]. The pathways represented in these databases are commonly used as a starting point (seed network) to analyze gene expression data and identify pathway activity using computational tools such as GSEA [4] and DAVID [5]. However, when an annotated pathway is used to analyze microarray gene expression data, the assumption is made that the ideal microarray derived network will be the same as that in the literature. This assumption may not hold since many pathways are defined based on observed protein-protein and protein-DNA interactions, metabolic fluxes, and subsets of particularly well-studied genes. Each of these factors may contribute to the substantial inconsistency between RNA-level microarray-based networks and currently defined pathways. Furthermore, the selected pathway representation may be incomplete and not include relevant regulator or effector molecules, thus necessitating computational prediction and subsequent validation. To address this issue, we introduce a method to systematically expand a pathway by identifying new genes that, from a gene expression perspective, better define the pathway itself.

Biological pathways have been constructed from the existing literature and annotation information using a wide range of methods [6], [7], [8], [9], [10], [11], [12], [13], [14]. One method of pathway reconstruction uses Bayesian networks (BNs) to learn and model relationships between variables (e.g., genes). Bayesian networks are graphical models that describe causal or apparently causal interactions between variables. In this study, a Bayesian network is defined as a set of interactions (edges or arrows) between variables (nodes) selected from a set of known pathway genes. High scoring BN topologies are learned from data based on scoring metrics such as the BDe scoring metric introduced by Cooper et al. in 1992 [15], that incorporates the joint probabilities for variables connected to one or more other variables. In this context, the Bayesian model is a multinomial model with a uniform Dirichlet prior. Bayesian networks such as these have been used to identify relationships from gene expression data [9], [16], protein-protein interactions[17], [18], and the regulation of phosphorylation states [19]. Due to their flexibility, reliability, ability to model multi-variable relationships, and human interpretability, Bayesian networks are well suited for network modeling using high-throughput data such as gene expression microarrays.

Networks learned from datasets such as gene expression data can be used to expand our knowledge about a known pathway, by independently testing the effects of added genes or variables on the overall scores of the corresponding expanded networks. A general network expansion framework to predict new components of a pathway was suggested in 2001 [20]. Many of the pathway expansion methods use correlation or Boolean functions [20], [21], [22], [23]. Compared to these methods, Bayesian network-based expansion methods provide distinct advantages, including prediction of both linear and nonlinear functions, identification of causal influences representing interactions among genes. Bayesian network-based expansion was also used for gene expression data analysis [24], [25]. However, these expansion approaches are module-based methods that focus on identifying modules (or groups) of additional genes to one gene [24] or a group of genes with a fixed topology [25]. The mRNA-based networks were also merged with protein data which often do not agree with each other [25]. The topology of the biological pathways may not be consistent with networks learned from transcriptional gene expression data obtained via DNA microarray studies [21].

We hypothesize that Bayesian networks derived from microarray gene expression data are largely consistent with known pathway models and can be used as a basis to predict novel factors that influence a given pathway. In this study, the hypothesis was examined using the *Escherichia coli* reactive oxygen species (ROS) pathway. Because *E. coli* and the ROS pathway had been well studied [26], [27], [28], [29], we were able to test the effectiveness of our network expansion algorithm and to assess the ability to reconstruct and expand an accepted pathway using microarray data. We identified many stress-related genes potentially involved in the ROS pathway and predicted their interactions with known ROS genes. Our prediction was confirmed experimentally for one example gene, *uspE*. Our single-gene expansion approach, termed ‘BN+1’, was successful in predicting unknown stress interactions that can be verified through experimental analysis, and could demonstrably be applied to other biological systems of interest.

## Results

**Figure 1:**
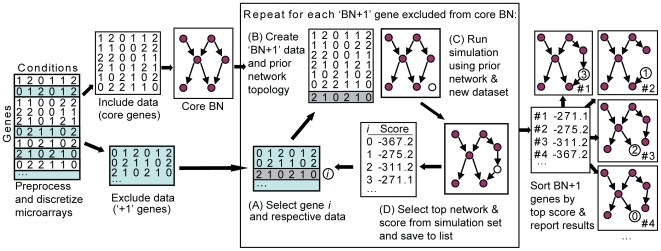
Schema for the BN+1 expansion algorithm. Bayesian networks are generated from discretized microarray data and ranked according to log posterior score. One of the top-scoring networks was selected as a core network for subsequent expansion. Each gene not included in the core network yet appearing in the microarray dataset was independently tested for its ability to acquire the best log posterior score versus the other tested expansion genes.

Below we describe the Bayesian network pathways identified from gene expression data, and the expansions to each network as predicted using the BN+1 algorithm (Figure 1).

### Microarray-Based Bayesian Network Overlapped with Known ROS Pathway

**Figure 2:**
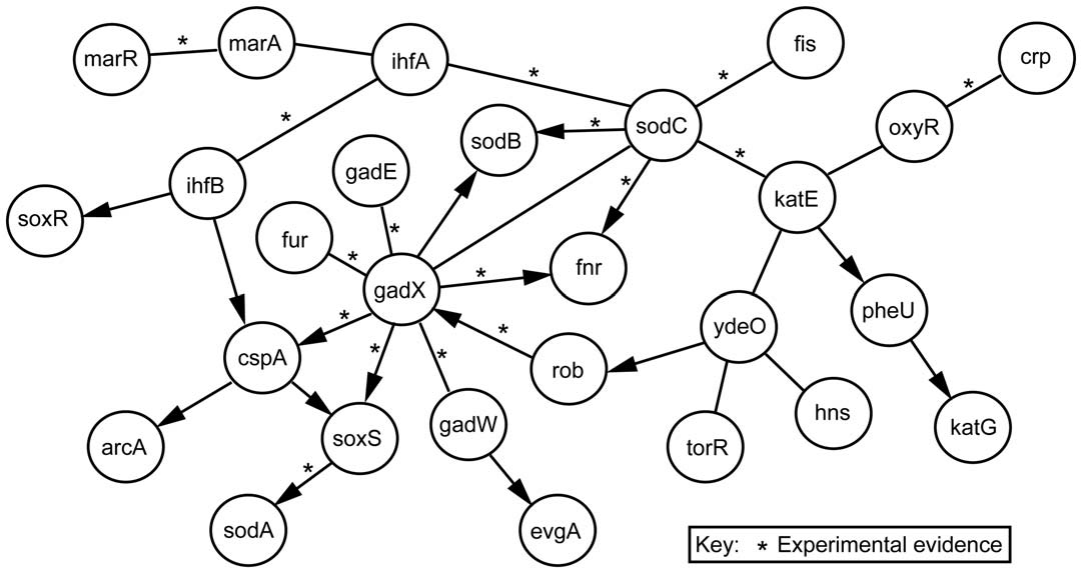
Consensus network for the ROS detoxification pathway based on gene expression data. Bayesian networks were generated using twenty-seven genes from the reactive oxygen species (ROS) detoxification pathway as variables or nodes and 305 gene expression microarray observations per variable. Edges which appear in the consensus and are supported by external data (e.g. EcoCyc, RegulonDB, and/or literature) are indicated (see Table S1).

Using a compendium of microarray gene expression data from the M3D database [30], networks were constructed for the 27 genes contained in the ROS pathway as defined by the EcoCyc database [1] (Figure 2). *E. coli* uses a complex detoxification pathway to protect against the oxidative stress posed by reactive oxygen species (ROS), including oxygen ions, free radicals, and peroxides [28]. The 27 genes identified in the EcoCyc ROS pathway include five ROS-processing enzymes (i.e., *katE*, *katG*, *sodA*, *sodB*, *sodC*) and 22 transcriptional factors that regulate transcription of these ROS-related enzymes. This *E. coli* expression dataset incorporates a variety of experimental conditions including time course studies, cell stress-inducing environments, over-expression, and single and double knockout strains. These conditions perturb the ROS pathway and provide a reasonable data set for the evaluation of our hypothesis. Our simulation results showed that more than one Bayesian network generated for the ROS pathway shared the same top posterior probability score. Therefore, a consensus network was derived using the 33 top networks that shared the best identical posterior probability. The consensus network contains all 27 genes from the original ROS detoxification list in EcoCyc.

A comparison of the consensus network to EcoCyc revealed that 29% of the edges in the consensus are supported by corresponding edges in EcoCyc [1] or RegulonDB [31]. However, inclusion of literature information in the comparison revealed that approximately 42% of the edges found in the consensus network were confirmed (Table S1). The difference suggests that some new literature results have not been collected in current databases such as EcoCyc and RegulonDB.

### BN+1 Pathway Expansions Predict ROS-Related Genes and Gene Interactions

An expansion algorithm termed BN+1 was developed to identify those genes that provide the best network score when added to an existing core network topology (Figure 1). This core network is a representative Bayesian network randomly selected from those top-scoring networks. Each gene not yet included in the core network is individually added to the set of variables for the Bayesian network simulation (hence Bayesian network plus one gene, or ‘BN+1’). The edges in the initial core network topology are used as a ‘structural prior’ or starting point, and are allowed to change over the course of the BN simulations. The added node is initially disconnected from the existing core network and can become connected to other variables over the course of the simulation. Those genes which best improve the network score when added to the existing core are expected to have the most direct biological influence and/or relevance to the core network genes.

**Figure 3:**
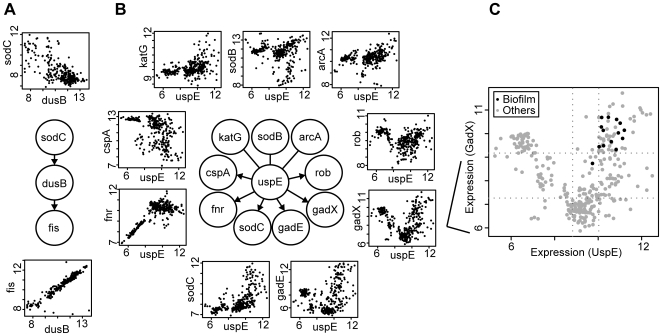
The genes *dusB*(A) and *uspE* (B) were the top results for the large network expansion. (C) Scatter plot for *uspE* versus *gadX* highlighting experiments with the word “biofilm” in the experiment title and/or description. High levels of *uspE* and *gadX* were observed for all conditions mapped to ‘biofilm’. The dotted lines indicate boundaries for binning used in network learning. A similar profile was shown for gadE (not shown).

The BN+1 expansion algorithm was used to identify additional potential members of the ROS detoxification pathway. The top-ranked results from these analyses are shown in Table 1. The algorithm identifies whether a gene is strongly associated with a particular network (e.g., the ROS detoxification pathway) and which genes in the network may influence or be influenced by the newly predicted gene. The predicted influences between core genes and the top “+1” genes (including *dusB* and *uspE*) identified by BN+1 expansion are shown in Figure 3.

**Table 1:** Top 10 genes identified by BN+1 expansion of the top Bayesian network.

Rank	Top BN+1 gene hits	Posterior BN score
1	***dusB*** (tRNA-dihydrouridine synthase B)	S = −8295.81
2	***fdhE*** (formate dehydrogenase formation protein)	S = −8298.44
3	***uspE*** (stress-induced protein);	S = −8310.63
4	***yohF*** (predicted oxidoreductase with NAD(P)-binding Rossman-fold domain)	S = −8312.24
5	***yncG*** (predicted enzyme);	S = −8313.04
6	***msyB*** (predicted protein);	S = −8318.20
7	***yedP*** (conserved protein);	S = −8320.30
8	***sra*** (30S ribosomal subunit protein S22)	S = −8323.97
9	***ydcK*** (predicted enzyme);	S = −8325.91
10	***ynhG*** (conserved protein);	S = −8326.20

Note that the numbers shown after gene names are negative logs of posterior probabilities for each top network containing the respective predicted gene.

Expansion of the core network revealed that many top predicted genes have known relationships with ROS and stress regulation (Table 1). The tRNA-dihydrouridine synthase B gene (*dusB* or *yhdG*) was predicted to be the top-scoring BN+1 gene and to interact with *fis* and *sodC* (Figure 3A). Fis is an important regulator of oxidative stress [32]. Because all of the known enterobacterial *fis* genes are preceded by *dusB* (also called *yhdG*) within the same operon [32], it is reasonable that *dusB* is positioned as a parent of *fis* in our prediction. The gene *dusB* is highly similar to *nifR3* [32], an element of the nitrogen regulatory system in bacteria [33]. A phylogenetic anlaysis of *fis* and *dusB* indicated that both genes were acquired by a lineage ancestral to γ-proteobacteria (including *E. coli*) from the *nifR3*-*ntrBC* operon of an ancestral α-proteobacterial lineage by lateral gene transfer [32]. Since *fis* is an important ROS regulator, it is likely that *dusB*, which was acquired together with *fis* and shares the same operon with *fis*, also plays an important role in ROS regulation. However, further experimental evidence is required to confirm the role of dusB in ROS regulation. Both *fis* and *sodC* are crucial to bacterial defense against the deleterious effects of reactive oxygen species (ROS) [34], [35]. The interaction between *sodC* and *dusB* is likely important for bacterial antioxidant reactions. The second top predicted gene *fdhE* encodes an *E. coli* formate dehydrogenase accessory protein that regulates the activity of catalytic sites of aerobic formate dehydrogenases and their redox activities [36]. A third gene, the universal stress protein *uspE*, is a known major regulator of motility factors and cell aggregation under stress conditions [37]. Several other predicted enzymes (*yncG* and *ydcK*) and proteins (*msyB*) found in the BN+1 search have no currently known functions related to the ROS pathway and stress response.

Pair-wise plots of the expression of BN+1 genes versus ROS pathway genes show simple (*dusB* vs *fis*, Figure 3A) or complex relationships (*uspE* vs. *gadX*, Figure 3B–C). The plots show that the relationships between these genes may be nonlinear. For example, a “V” shaped pattern is observed between the expression profiles of *gadX* and *uspE*, where *gadX* is down-regulated at moderate levels of *uspE* and up-regulated in either increased or decreased levels of *uspE* (Figure 3C). This special non-linear gene interaction pattern was not clearly demonstrated in a traditional hierarchical clustering heatmap (Figure S1). Gene *gadX* is a transcriptional regulator of glutamic acid decarboxylase system, which enables *E. coli* to overcome acidic stress, while *uspE* is a universal stress-induced protein. A term enrichment method was generated to identify words that are preferentially grouped and reflect most significant features of the interactions between two genes (e.g., *gadX* and *uspE*) as predicted by our BN method.

Based on our term enrichment analysis of *gadX* and *uspE*, one term that clustered the data particularly well was “biofilm”, which was demonstrated in the annotated scatter plot (Figure 3). High expression of *gadX* was correlated with high expression of *uspE* in biofilms. Biofilms are aggregates of microorganisms that attach to and grow on a surface in contact with liquid, such as water or media. Induced expression of stress response genes, e.g., a universal stress regulater *uspA*, was a general feature of biofilm growth [38], [39]. In fact, the biofilm microarray data used in the term enrichment were obtained from two studies. One study analyzed stress-oriented gene expression profiles of *E. coli* biofilm at various time points [40]. A second biofilm microarray study examined biofilm responses to acid resistance and oxidative stress using wild type and single gene knockout mutant strains of *E. coli* [41]. Our combined analysis of microarray gene expression and term enrichment indicated that *uspE* and *gadX* were both up-regulated in many samples (chips) where ‘biofilm’ was mentioned in the sample title and/or description (Figure 3B–C). These suggested a potential role of the *uspE* and *gadX* in the formation of *E. coli* biofilm.

To further evaluate the interactions between *uspE* and *gadX* and their regulatory roles in ROS stress and biofilm formation, several wet-lab experiments were conducted as described below.

### Confirmation of the Involvement of Gene *uspE* and *gadX* in ROS Network

**Figure 4:**
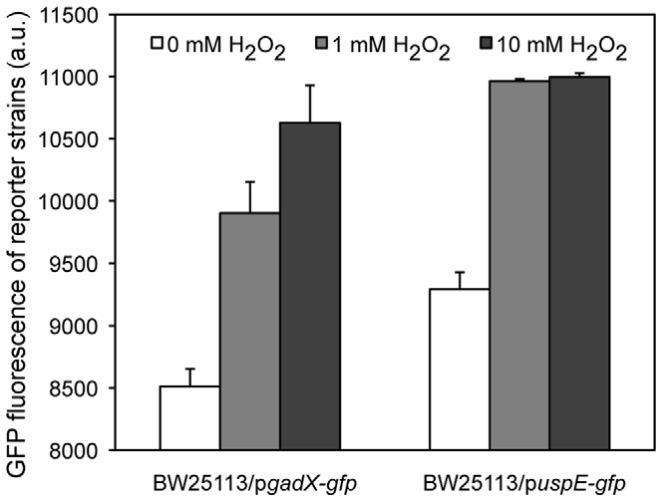
Expression profiles of *E. coli gadX* and *uspE* upon exposure to hydrogen peroxide. Change of GFP fluorescence of two reporter strains *E. coli* BW25113/p*gadX-gfp* and BW25113/p*uspE-gfp* upon exposure to 0 mM, 1 mM and 10 mM hydrogen peroxide for 20 min. Cells were cultured in LB broth at 30°C overnight and re-suspended in 1×PBS. Different concentration of hydrogen peroxide was added into three aliquots for 20 min before cell density (OD) and fluorescence intensity were measured. Presented GFP fluorescence for each sample was normalized to OD. Error bar indicated standard deviation from two replicated cell cultures.

Regulation of gene expression involved in the ROS network upon exposure to ROS was widely reported [26], [27], [28], [29], [34], [35], [37]. Hydrogen peroxide is one of the commonly used ROS. To test the involvement of *uspE* and *gadX* in the ROS network, gene expressions of *uspE* and *gadX* were monitored after exposure of two reporter strains, *E. coli* BW25113/p*gadX-gfp* and BW25113/p*uspE-gfp*, to hydrogen peroxide. GFP fluorescence of the reporter strain indicated expression of the corresponding gene. Compared to a control not exposed to hydrogen peroxide, GFP fluorescence of both reporter strains significantly increased in exposure to both 1 mM and 10 mM hydrogen peroxide (Figure 4). This indicated that expression of *gadX* or *uspE* was up-regulated upon exposure to hydrogen peroxide. It confirmed that both genes were involved in the ROS network as predicted by our BN+1 method.

### Confirmation of Interactions between *uspE* and *gadX*

**Figure 5:**
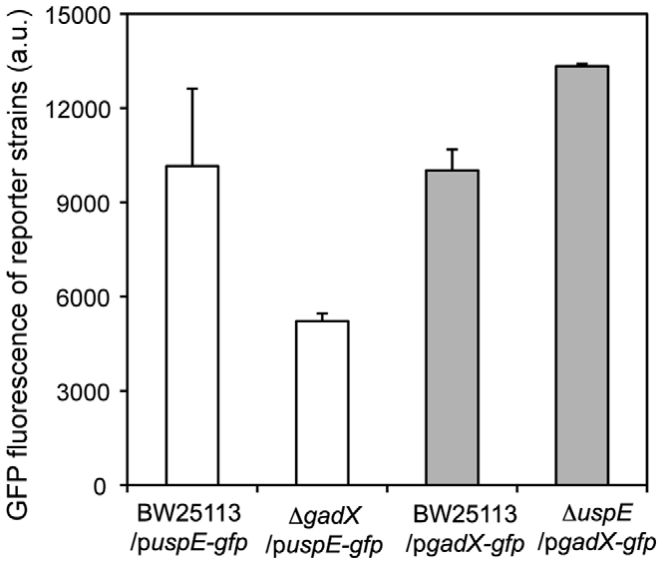
Analyses of the *gadX*-*uspE* interaction through knockout studies. GFP fluorescence of wild type *E. coli* BW25113 and single gene knockout mutant Δ*gadX* carrying the reporter plasmid p*uspE-gfp*, and wild type *E. coli* and single gene knockout mutant Δ*uspE* carrying the other reporter plasmid p*gadX-gfp.* Cells of each reporter strain were cultured in LB broth at 30°C overnight and re-suspended in 1×PBS before cell density (OD) and fluorescence intensity were measured. GFP fluorescence for each strain was normalized to the OD value. Error bars indicated standard deviations from two replicated cultures each with four replicate readings.

To measure the interactions between gene *uspE* and *gadX*, two mutant reporter strains, Δ*uspE/*p*gadX-gfp* and Δ*gadX/*p*uspE-gfp* were generated with *gadX* and *uspE* deleted, respectively. The two mutants provide a way to monitor the effect of deleting one gene on the expression of the other gene. Specifically, GFP fluorescence of mutant reporter strains Δ*gadX/*p*uspE-gfp* and Δ*uspE/*p*gadX-gfp* were compared to fluorescence of their corresponding wild type reporter strains, BW25113/p*uspE-gfp* and BW25113/p*gadX-gfp*, respectively. The results showed that expression of gene *uspE* was significantly decreased to half level when gene *gadX* was knocked out, while *gadX* expression was significantly increased if gene *uspE* was knocked out (p-value<0.0001) (Figure 5). The results suggested that *gadX* induced the expression of gene *uspE*, while *uspE* may repress the expression of gene *gadX*. The fact that gene *gadX* and *uspE* influenced the expression of each other confirmed our prediction of the influences between the two genes and further refined their biological interactions.

**Figure 6:**
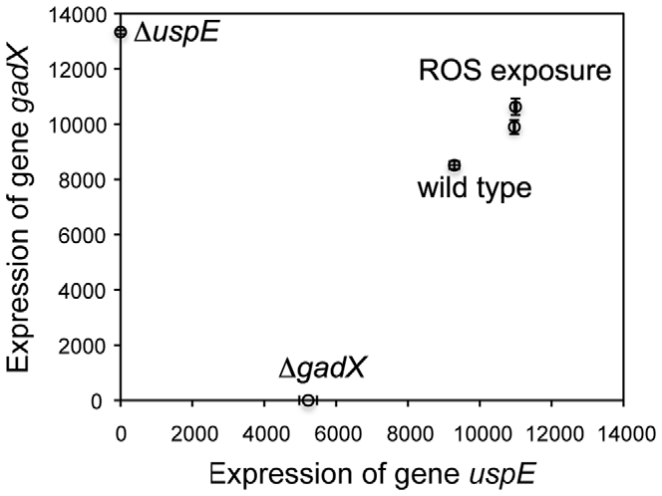
Summary of *gadX* and *uspE* gene expression under various experimental conditions. Plot of the expressions of *gadX* (x-axis) and *uspE* (y-axis) against each other in different strain backgrounds and tested experimental conditions. The expression of *gadX* or *uspE* was represented by the GFP fluorescence of the reporter strains carrying the respective reporter plasmids p*gadX*-*gfp* or p*uspE*-*gfp*. The strain background or experimental conditions were noted by the data. Expression of gene *uspE* or *gadX* was assumed as zero in its single gene mutant Δ*uspE* or Δ*gadX*, respectively. Wild type strain was used in the ROS exposure experiments using 1 mM and 10 mM hydrogen peroxide. Error bars indicate standard deviation from replicates.

GFP fluorescence of different *E. coli* strains (wild type or single gene knockout mutant strains) carrying reporter plasmids p*gadX-gfp* or p*uspE-gfp* indicated expression of the gene *gadX* or *uspE* in these strains, respectively, under the tested experimental conditions. The expressions of the gene *gadX* and *uspE* (GFP fluorescence of the different *E. coli* strains carrying the two reporter plasmids) under different tested conditions in the above confirmation experiments were plotted against each other (Figure 6). This plot demonstrated a roughly “V” shaped pattern similar to that shown in the plot of gene expression data pooled from microarray studies (Figure 3C).

### Confirmation of the Involvement of Gene *uspE* and *gadX* on Biofilm Formation

**Figure 7:**
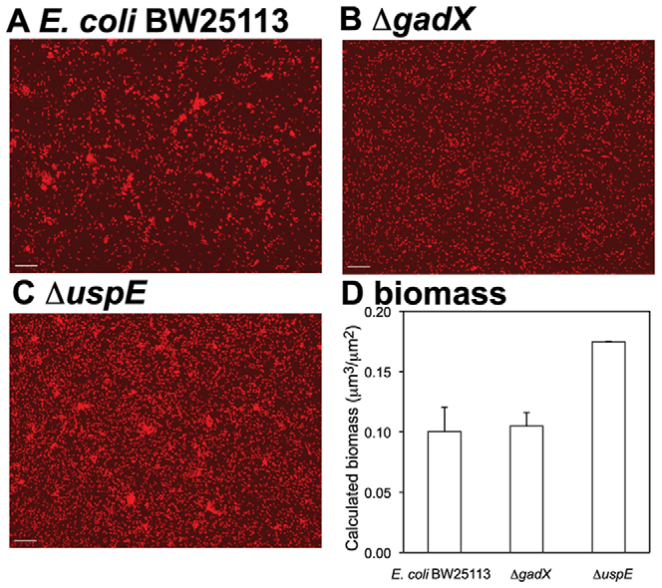
The effect of *gadX* and *uspE* on *E. coli* biofilm formation. Fluorescent micrograph of biofilms formed by (A) wild type *E. coli* BW25113, (B) single gene knockout mutant Δ*gadX*, and (C) single gene knockout mutant Δ*uspE.* Biomass of biofilms formed by each strain was calculated (D) using the software COMSTAT. Biofilms were formed on glass bottom of 24-well plates for 3 h after inoculation. Suspended cells were gently removed. Biofilms were gently washed with PBS twice and stained with Syto 60 for 10 min before microscopic examination. Images were taken from randomly chosen spots near the center of the well. Error bar in the calculated biomass was standard deviation from three stacks of images. Scale bar = 10 µm.

Biofilm cells response to a wide range of stresses [42]. Many ROS related genes have been found to be up-regulated in biofilms [38], [39]. For example, *arcA* (a gene in our ROS core network) was reported to be important for competitiveness in *E. coli* biofilms [42]. Our term enrichment method identified “biofilms” as a significantly enriched term associated with the gene pair of *uspE* and *gadX*. Those microarray chips containing “biofilms” in their experimental descriptions frequently show high expressions of both *uspE* and *gadX* as demonstrated in Figure 3. To test the involvement of gene *uspE* and *gadX* in biofilm formation, initial biofilm formation (3 h attachment and growth) on glass surface by wild type *E. coli* BW25113 and single gene knockout mutants, Δ*gadX* or Δ*uspE*, was examined using confocal laser scanning microscopy (CLSM). The structure of biofilm formation was measured by a typical *en face* image of biofilms of each strain (Figure 7A–C). The extent of biofilm formation was quantified using biofilm biomass (Figure 7D). The results showed that biofilms formed by the Δ*uspE* strain contained higher biomass than biofilms formed by the wild type strain. The Δ*gadX* biofilm had similar biomass but different structures compared to biofilms by wild type *E. coli* strain. Microcolonies were observed in biofilms of wild type strain (Figure 7A), while biofilms of Δ*gadX* were mostly single layer of attached cells at this observation stage (Figure 7B). The observed difference in biofilm biomass and structure in biofilms formed by the *uspE* or *gadX* knockout mutant and wild type strain indicates that both gene *uspE* and gene *gadX* were involved in biofilm formation by *E. coli*.

In summary, the BN+1 algorithm predicted that the *uspE* gene was a new gene in the ROS network and that the *uspE* gene interacts with many ROS-related genes including *gadX*. Our further text mining analysis predicted that *gadX* and *uspE* gene may be important in biofilm formation. These three predictions were then successfully verified in experiments.

## Discussion

In this study, we addressed two questions: (1) Does a microarray-based Bayesian network reconstruction match with the known pathway from the literature and existing database? (2) Is a network expansion approach such as BN+1 useful in predicting new, biologically significant genes?

For the first question, our studies indicated that the microarray-based Bayesian network reconstruction did not always agree with the known pathway from the literature and databases. Our studies on the *E. coli* ROS pathway indicated that the network reconstructed by our Bayesian network overlaps at 29% with the known ROS pathway network in EcoCyc and RegulonDB (Table S1). A 42% agreement was achieved when more evidences from the literature search was included. Inclusion of RegulonDB and literature resources made our comparison more comprehensive. The reason for the large mismatch is probably due to the fact that microarray-based transcriptional data may not reflect the complex biological pathways which involve complex interactions of genes in the protein, RNA, and DNA levels [43]. However, the Bayesian networks built from microarray gene expression data are transcriptional regulatory models that are predicted to reflect the complex ROS pathway.

For the second question, the BN+1 expansion algorithm was found to successfully predict biologically significant genes to the ROS network that were further experimentally verified. Gene *uspE* was one of the top list genes selected by the BN+1 algorithm. Its up-regulation in response to the exposure of hydrogen peroxide suggested that this gene was probably involved in the ROS network, along with the ROS-related gene *gadX* (Figure 4). Hierarchical clustering of the *uspE* gene showed a different connectivity pattern in the dendrogram for genes than the Bayesian network, suggesting that the Bayesian network identified a non-traditional (*e.g.* nonlinear) relationship between the genes. Furthermore, the BN+1 algorithm suggested where the new genes could participate in the pathway, and in some cases the model even differentiated between the parents and children genes of a new gene (Figure 3–4). Specifically, the BN+1 algorithm found the “V” shape relationships between expressions of genes, e.g., *gadX* and *uspE,* which would not have been identified using traditional clustering approaches. The interaction between gene *gadX* and *uspE* was also confirmed experimentally. Expression of one gene was significantly affected when the other gene was knocked out from the wild type *E. coli* strain (Figure 5). Plot of the expression of *gadX* and *uspE* against each other under different tested experimental conditions showed a similar “V” shaped pattern (Figure 6), which was in agreement with the finding using the BN+1 algorithm although the expression data from the experimental study were at the translational level.

The term enrichment algorithm successfully identified experimental conditions in which genes might be involved and biologically related with each other. In this study, genes *uspE* and *gadX* were founded to be both up-regulated in the growth of biofilms. The involvement of the two genes in biofilms was confirmed by the fact that single gene knockout mutant strains Δ*gadX* and Δ*uspE* showed difference in the biofilm formation, either biomass or structures, as compared to the *E. coli* wild type strain (Figure 7). Experimental confirmation of predicted term enrichment results indicates that term enrichment algorithm is a useful method to identify experimental conditions in which gene relationship may take place, or to propose additional areas of investigation. Performance of the term enrichment approach likely depends upon the quality of the experimental descriptions provided by researchers available from the M3D database. The approach may perform better with controlled term or concept vocabularies, or could be further tested with Gene Ontology (GO) terms and other information in future studies.

Bayesian networks can be used to expand a pathway network based on microarray gene expression data. The BN+1 method expands a top Bayesian network by adding one gene at a time and running it iteratively based on microarray gene expression data. The BN+1 expansion algorithm showed the ability to predict important factors for a pathway network from thousands of genes in a microarray study. The BN+1 approach is a generalized method to refine and expand biological pathways. Although a ROS pathway in *E. coli* was shown in this study, the BN+1 algorithm can readily be applied to other organisms, pathways, and data types. We also plan to develop a BN+1 expansion method based on dynamic Bayesian network analysis [44]. Furthermore, the term enrichment-based identification of experimental conditions in the context of binned data for BN analysis can provide beneficial information in the interpretation of predicted expansion genes.

## Methods

### Data Preprocessing

A compilation dataset comprising 305 gene expression microarray observations and 4,217 genes from *Escherichia coli* MG1655 was obtained from the M3D database [30]. A coefficient of variation threshold (c.v. ≥1.0) was used to select 4,205 genes for analysis. Twenty-seven genes were identified from the EcoCyc ROS detoxification pathway (downloaded on March 26, 2008) and matched to unique features found in 305 available gene expression microarray chips. Expression profiles for each gene were discretized using a maximum entropy approach that uses three equally-sized bins (q3 quantization).

### Learning Bayesian Network Pathway Models

Given the set of 27 genes, Bayesian network analysis was used to learn the structure of the model which served as our core starting topology. To maximize the network search space, 4000 independent simulations with random starts were used to search 2.5×10^7^ networks per start for a total of 1×10^11^ networks. The five top networks were saved from each run, thereby generating a final list of 20,000 top-scoring networks. These networks were used to estimate the posterior distribution. During the search, each network was scored using log of the BDe score [15], [45] which is the natural log of posterior probability (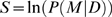). Here 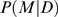 is defined as:
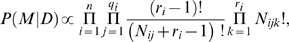
where *n* is the number of variables, *q_i_* is the number of parent configurations for given variable *i*, *r_i_* is the arity of variable *i*, *N_ij_* is the number of observations with selected parent configuration *q_i_*, *N_ijk_* is the number of observations of child in state *k* with parent configuration *q_i_* [15]. The calculation of this score was implemented using the software package BANJO [46].

A consensus network was generated using 33 networks which shared the maximum or best log posterior score (ln(P(D|M)). Specifically, directed edges in the consensus networks represent those edges that appear with 100% frequency in one direction in all of these top networks. Undirected edges represent those edges appearing 100% of the time in both directions in all stored networks (Figure 2).

### Network Expansion Using BN+1

To expand an existing network, a top network used to generate the consensus network was selected as a starting topology for the BN+1 algorithm (Figure 1). A set of 4,178 genes (4,205–27), not included in the top BN, were tested for their ability to improve score of the initial core BN when added to the initial gene set. In each iteration of the BN+1 simulation, the current BN+1 gene was added to the original data file. This was followed by a simulated annealing search of 1×10^7^ networks for the top network expansion. Although the top network was selected as a starting point or seed, during the learning round all edges could be modified such that the addition of genes could change the backbone structure of the resulting model (i.e., unfixed structural prior). Genes were sorted based on their log posterior scores. BN+1 searches for each of the top 200 genes recovered from the initial top network were rerun (2.5×10^7^ networks/simulation with 150 replicate simulations) to allow sufficient convergence.

All calculations, including the network expansion, were implemented in a publicly available, internally developed software program MARIMBA (http://marimba.hegroup.org/).

### Term Enrichment for Identifying Relevant Experimental Observations

A term enrichment program was developed to identify which descriptive terms in the experimental conditions show significant enrichment in selected regions of the microarray data. A ‘term’ here is defined as any individual word appearing in the names or descriptions for each microarray sample. For two selected genes, a p-value was introduced to determine the chance of observing a selected term in a selected bin. The p-value was calculated using the Fisher's exact test for appearance of ‘term’ and ‘non-term’ data observations in a specific bin [47]. The bins used for microarray BN analysis were adopted in this term enrichment analysis. For example, the q3 quantization was used for the expression levels of *gadX* and *uspE*.

### Experimental Validation of our Prediction Using Gene *uspE* as an Example

#### Strains and cell cultures

*E. coli* K-12 wild type strain BW25113 and single gene knockout mutant strains (Δ*uspE* and Δ*gadX*) were obtained from the KEIO collection [48]. Cell cultures were inoculated from single colonies on Luria broth (LB) agar plates, supplied with 20 µg/ml tetracycline, 30 µg/ml chloramphenicol, or 20 µg/ml kanamycin (Sigma-Aldrich, St. Louis, MO) appropriately. Planktonic cell cultures were grown in LB overnight with a continue shaking (250 rpm) at 30°C.

#### Plasmids construction

Plasmids pUA66 or pUA139 carrying a *gfp*-fusion with the promoter of *gadX* or *uspE* were extracted from corresponding strains in the promoter library PEC3877 (Open Biosystems, Huntsville, AL) [49]. A tetracycline resistance gene (*tetR*) was cloned from pMP4655 [50] using the set of forward and backward primers, ACATGGCTCTGCTGTAGTGA and CGACATGTCGTTTTCAGAAG respectively. Clone *tetR* was inserted in the AfeI (NEB, Ipswich, MA) digestion site of the reporter plasmids to acquire two reporter plasmids named as p*gadX-gfp* and p*uspE-gfp*. The two plasmids were individually transformed into *E. coli* BW25113 strains by electroporation (Bio-Rad, Hercules, CA). Single colonies of *E. coli* were acquired on selective agar plates containing 20 µg/ml tetracycline. Reporter plasmids p*gadX-gfp* and p*uspE-gfp* were then extracted from single colonies of *E. coli*, and then transformed into wild type *E. coli* strains and single gene knockout mutant Δ*uspE* and Δ*gadX*, respectively, to get totally four reporter strains.

#### Gene expression analysis

Planktonic cultures of the four reporter strains, wild type *E. coli* BW25113/p*gadX-gfp*, BW25113/p*uspE-gfp*, Δ*uspE*/p*gadX-gfp*, and Δ*gadX*/p*uspE-gfp*, were washed and re-suspended in phosphate buffered saline (PBS). Cell growth (optical density OD at 600 nm) and fluorescence intensity of tagged GFP were measured in a plate-reader (Bio Tek, Winooski, VT). Normalized fluorescence to OD was calculated and used to indicate expression of gene *gadX* and gene *uspE* in wild type *E. coli* as well as in single gene knockout mutants. Two independent cultures were performed, each with three replicates of measurement.

Planktonic cultures of *E. coli* BW25113/p*gadX-gfp* and BW25113/p*uspE-gfp* were used to monitor expression of gene *gadX* and gene *uspE* in response to the exposure of hydrogen peroxide. Final concentration of 1 mM and 10 mM hydrogen peroxide (Fisher Scientific, Pittsburgh, PA) was added into PBS re-suspended *E. coli* cells for 20 min. OD and GFP fluorescence intensity were measured in the plate-reader, using the same *E. coli* strains without exposure to hydrogen peroxide as controls. OD adjusted GFP fluorescence intensity was used to indicate gene expression of *gadX* or *uspE*.

GFP fluorescence of different *E. coli* strains (wild type or single gene knockout mutant strain) carrying reporter plasmids p*gadX-gfp* or p*uspE-gfp* was summarized in a plot (Figure 6), assuming that expression of the *gadX* gene and the *uspE* gene were zero in its corresponding knockout mutant, respectively.

#### Biofilm cultures and analysis

Planktonic cultures of wild type *E. coli* and single gene knockout mutant Δ*gadX* and Δ*uspE* were acquired from overnight cultures in 0.1×LB. Cultures were mixed with the same volume of fresh 0.1×LB before second culture at 30°C for 4 hours. New cultures were added into 24-well glass bottom plates (1 ml/well, MatTek, Ashland, MA) and kept static for three hours at room temperature to allow cells to attach onto the surface and form biofilms. Supernatant was gently removed and biofilms were washed with PBS twice. Biofilm cells were stained with 5 µm Syto 60 (Invitrogen, Carlsbad, CA) for 10 min. Biofilms were imaged randomly across the surface in the center of each well with a confocal laser scanning microscopy equipped with the software FluoView 300 (Olympus, Center Valley, PA). Biomass of biofilms was calculated using the program COMSTAT [51].

## Supporting Information

Figure S1 Heatmap of gene expression profiles of all core genes and the predicted *uspE* gene. This hierarchical clustering was generated using a Manhattan distance metric and average clustering via the Heatplus module in R.
(0.24 MB DOC)

Table S1 Database and literature evidence to support predicted Bayesian network interactions. Directed (→) and undirected (-) edges are shown for each level of consensus in the BN consensus networks.
(0.12 MB DOC)
