# Supplementary material for: Bayesian Network Expansion Identifies New ROS and Biofilm Regulators
Source: PLoS One. 2010 Mar 3;5(3):e9513. doi: 10.1371/journal.pone.0009513 (PMC2831072; doi:10.1371/journal.pone.0009513)
Supplement: Figure S1 — Heatmap of gene expression profiles of all core genes and the predicted uspE gene. This hierarchical clustering was generated using a Manhattan distance metric and average clustering via the Heatplus module in R. (0.24 MB DOC) [file pone.0009513.s001.doc]

**Figure S1**. Heatmap of gene expression profiles of all core genes and the predicted *uspE* gene. This hierarchical clustering was generated using a Manhattan distance metric and average clustering via the Heatplus module in R.

**Reference:**

Team, R.D.C., *R: A Language and Environment for Statistical Computing*. 2009, Vienna, Austria.
