# Supplementary material for: Bayesian Network Expansion Identifies New ROS and Biofilm Regulators
Source: PLoS One. 2010 Mar 3;5(3):e9513. doi: 10.1371/journal.pone.0009513 (PMC2831072; doi:10.1371/journal.pone.0009513)
Supplement: Table S1 — Database and literature evidence to support predicted Bayesian network interactions. Directed (→) and undirected (-) edges are shown for each level of consensus in the BN consensus networks. (0.12 MB DOC) [file pone.0009513.s002.doc]

**Table S1.** Database and literature evidence to support predicted Bayesian network interactions. Directed (🡪) and undirected (–) edges are shown for each level of consensus in the BN consensus networks.

| **BN** | **Is edge present in selected network?** | | | **Literature support** |
| --- | --- | --- | --- | --- |
| **Relationship** | **Present in consensus?** | **Present in EcoCyc?** | **Present in RegulonDB?** | **Strong support?** |
| *marR – marA* | yes | yes | yes | Yes: regulon [1,2] |
| *marA – ihfA* | yes | no | no |  |
| *ihfA – ihfB* | yes | yes | yes | Yes: autoregulation by *ihf* [3] |
| *ihfA – sodC* | yes | yes | no |  |
| *ihfB* 🡪 *cspA* | yes | no | no |  |
| *ihfB* 🡪 *soxR* | yes | no | no |  |
| *torR – ydeO* | yes | no | no |  |
| *hns – ydeO* | yes | no | no |  |
| *ydeO* 🡪 *rob* | yes | no | no |  |
| *ydeO – katE* | yes | no | no |  |
| *oxyR – crp* | yes | yes | yes | Yes: *oxyR* upregulated by *crp* [4] |
| *oxyR – katE* | yes | no | no |  |
| *fis – sodC* | yes | no | no | Yes: *sodC* regulated by *fis* [5] |
| *katE* 🡪 *pheU* | yes | no | no |  |
| *pheU* 🡪 *katG* | yes | no | no |  |
| *katE – sodC* | yes | no | no | Yes: genomic region regulated by *rpoS* [6] |
| *sodC – gadX* | yes | no | no |  |
| *rob* 🡪 *gadX* | yes | no | no | Yes: co-regulation by *rpoS*, feed forward loop [7] |
| *sodC* 🡪 *fnr* | yes | no | no | Yes: *sodC* repressed anaerobically by *fnr* [8] |
| *sodC* 🡪 *sodB* | yes | yes | no | Yes: Co-regulated by *fis* [5], *fur* [9] |
| *gadX* 🡪 *cspA* | yes | no | no | Yes: Two-step flux process: *cspA*->*hns*->*gadX* [10] |
| *gadX – fur* | yes | no | no | Possible: negative correlation observed for c-di-GMP [11,12] |
| *gadX* 🡪 *soxS* | yes | no | no | Possible: negative correlation observed for c-di-GMP [12] |
| *gadX – gadW* | yes | yes | yes | Yes: direct regulation [13,14] |
| *gadX – gadE* | yes | yes | yes | Yes: direct regulation [13,14] |
| *gadX* 🡪 *fnr* | yes | yes | yes | Possible: predicted binding site for *fnr* to repress *gadX* [15] |
| *gadX* 🡪 *sodB* | yes | no | no |  |
| *cspA* 🡪 *arcA* | yes | no | no |  |
| *cspA* 🡪 *soxS* | yes | no | no |  |
| *soxS* 🡪 *sodA* | yes | yes | yes | Yes: *soxS* regulates *sodA* [16] |
| *gadW* 🡪 *evgA* | yes | no | no |  |
| **Positive (“yes”)**  **Counts** | **31** | **Supported consensus edges: 13/31 = 42%** | | |

**References for Supplemental Table 1:**

1. Ariza RR, Cohen SP, Bachhawat N, Levy SB, Demple B (1994) Repressor mutations in the marRAB operon that activate oxidative stress genes and multiple antibiotic resistance in Escherichia coli. J Bacteriol 176: 143-148.

2. Cohen SP, Hachler H, Levy SB (1993) Genetic and functional analysis of the multiple antibiotic resistance (mar) locus in Escherichia coli. J Bacteriol 175: 1484-1492.

3. Bykowski T, Sirko A (1998) Selected phenotypes of ihf mutants of Escherichia coli. Biochimie 80: 987-1001.

4. Gonzalez-Flecha B, Demple B (1997) Transcriptional regulation of the Escherichia coli oxyR gene as a function of cell growth. J Bacteriol 179: 6181-6186.

5. Kelly A, Goldberg MD, Carroll RK, Danino V, Hinton JC, et al. (2004) A global role for Fis in the transcriptional control of metabolism and type III secretion in Salmonella enterica serovar Typhimurium. Microbiology 150: 2037-2053.

6. Weber H, Polen T, Heuveling J, Wendisch VF, Hengge R (2005) Genome-wide analysis of the general stress response network in Escherichia coli: sigmaS-dependent genes, promoters, and sigma factor selectivity. J Bacteriol 187: 1591-1603.

7. Ma HW, Kumar B, Ditges U, Gunzer F, Buer J, et al. (2004) An extended transcriptional regulatory network of Escherichia coli and analysis of its hierarchical structure and network motifs. Nucleic Acids Res 32: 6643-6649.

8. Gort AS, Ferber DM, Imlay JA (1999) The regulation and role of the periplasmic copper, zinc superoxide dismutase of Escherichia coli. Mol Microbiol 32: 179-191.

9. Niederhoffer EC, Naranjo CM, Bradley KL, Fee JA (1990) Control of Escherichia coli superoxide dismutase (sodA and sodB) genes by the ferric uptake regulation (fur) locus. J Bacteriol 172: 1930-1938.

10. Martinez-Antonio A, Janga SC, Thieffry D (2008) Functional organisation of Escherichia coli transcriptional regulatory network. J Mol Biol 381: 238-247.

11. Chen Z, Lewis KA, Shultzaberger RK, Lyakhov IG, Zheng M, et al. (2007) Discovery of Fur binding site clusters in Escherichia coli by information theory models. Nucleic Acids Res 35: 6762-6777.

12. Mendez-Ortiz MM, Hyodo M, Hayakawa Y, Membrillo-Hernandez J (2006) Genome-wide transcriptional profile of Escherichia coli in response to high levels of the second messenger 3',5'-cyclic diguanylic acid. J Biol Chem 281: 8090-8099.

13. Mates AK, Sayed AK, Foster JW (2007) Products of the Escherichia coli acid fitness island attenuate metabolite stress at extremely low pH and mediate a cell density-dependent acid resistance. J Bacteriol 189: 2759-2768.

14. Richard H, Foster JW (2007) Sodium regulates Escherichia coli acid resistance, and influences GadX- and GadW-dependent activation of gadE. Microbiology 153: 3154-3161.

15. Constantinidou C, Hobman JL, Griffiths L, Patel MD, Penn CW, et al. (2006) A reassessment of the FNR regulon and transcriptomic analysis of the effects of nitrate, nitrite, NarXL, and NarQP as Escherichia coli K12 adapts from aerobic to anaerobic growth. J Biol Chem 281: 4802-4815.

16. Martin RG, Bartlett ES, Rosner JL, Wall ME (2008) Activation of the Escherichia coli marA/soxS/rob regulon in response to transcriptional activator concentration. J Mol Biol 380: 278-284.
